# Nanotechnological Strategies for Osteoarthritis Diagnosis, Monitoring, Clinical Management, and Regenerative Medicine: Recent Advances and Future Opportunities

**DOI:** 10.1007/s11926-020-0884-z

**Published:** 2020-04-04

**Authors:** Reza Mohammadinejad, Milad Ashrafizadeh, Abbas Pardakhty, Ilona Uzieliene, Jaroslav Denkovskij, Eiva Bernotiene, Lauriane Janssen, Gabriela S. Lorite, Simo Saarakkala, Ali Mobasheri

**Affiliations:** 1 Pharmaceutics Research Center, Institute of Neuropharmacology, Kerman University of Medical Sciences, Kerman, Iran; 2 Department of Basic Science, Faculty of Veterinary Medicine, University of Tabriz, Tabriz, Iran; 3 Department of Regenerative Medicine, State Research Institute Centre for Innovative Medicine, Santariskiu 5, LT-08406 Vilnius, Lithuania; 4 Microelectronics Research Unit, Faculty of Information Technology and Electrical Engineering, University of Oulu, PL 4500, 3FI-90014 Oulu, Finland; 5 Department of Diagnostic Radiology, Oulu University Hospital, Oulu, Finland; 6 Research Unit of Medical Imaging, Physics and Technology, Faculty of Medicine, University of Oulu, Oulu, Finland; 7 Centre for Sport, Exercise and Osteoarthritis Versus Arthritis, Queen’s Medical Centre, Nottingham, UK; 8 Sheik Salem Bin Mahfouz Scientific Chair for Treatment of Osteoarthritis with Stem Cells, King AbdulAziz University, Jeddah, Saudi Arabia; 9 University Medical Center Utrecht, Department of Orthopedics and Department of Rheumatology & Clinical Immunology, 508 GA Utrecht, The Netherlands

**Keywords:** Nanotechnology, Osteoarthritis, Cartilage, Diagnostic, Regenerative medicine

## Abstract

**Purpose of Review:**

In this review article, we discuss the potential for employing nanotechnological strategies for the diagnosis, monitoring, and clinical management of osteoarthritis (OA) and explore how nanotechnology is being integrated rapidly into regenerative medicine for OA and related osteoarticular disorders.

**Recent Findings:**

We review recent advances in this rapidly emerging field and discuss future opportunities for innovations in enhanced diagnosis, prognosis, and treatment of OA and other osteoarticular disorders, the smart delivery of drugs and biological agents, and the development of biomimetic regenerative platforms to support cell and gene therapies for arresting OA and promoting cartilage and bone repair.

**Summary:**

Nanotubes, magnetic nanoparticles, and other nanotechnology-based drug and gene delivery systems may be used for targeting molecular pathways and pathogenic mechanisms involved in OA development. Nanocomposites are also being explored as potential tools for promoting cartilage repair. Nanotechnology platforms may be combined with cell, gene, and biological therapies for the development of a new generation of future OA therapeutics.

**Figure:**
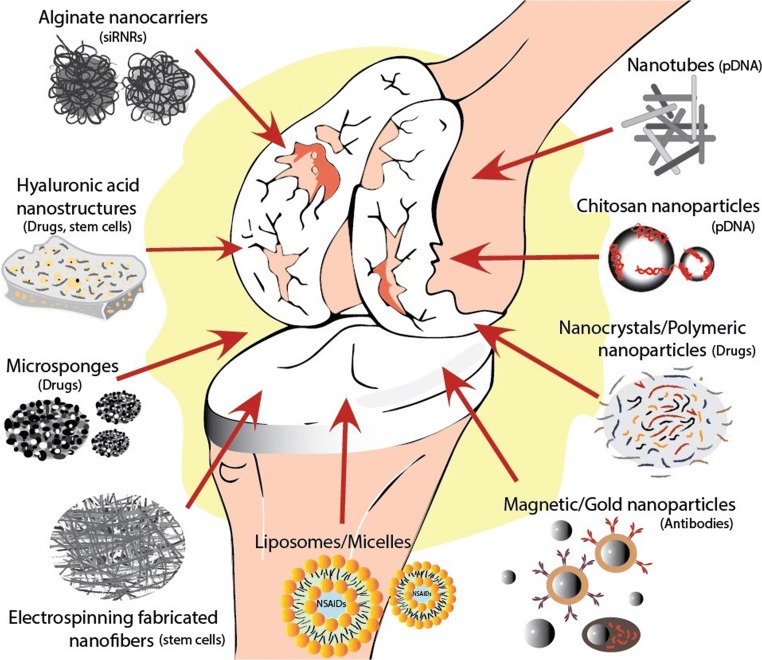
Graphical Abstract

## Introduction

Significant progress has been made in recent years in nanotechnology and nanomedicine. Nanotechnologies are used to deliver anticancer therapeutics, to perform minimally invasive image-guided delivery of plasmids and non-coding RNAs [1], and to facilitate the targeted delivery of conventional and biological drugs [2]. The main benefit of employing nanocarriers in the therapeutics arena is to achieve targeted delivery using the optimum drug dosage, extend drug circulation, reduce side effects, and decrease the likelihood of developing drug resistance. Nanotechnologies provide new platforms for achieving sustained drug release, preventing “burst release” and countering drug resistance.

Currently, nanoparticles (NPs) are the most innovative biomaterials for potential diagnosis and management of osteoarthritis (OA) [3–6]. Nanomaterials such as liposomes, micelles, carbon nanoallotropes, and quantum dots are described as particles with sizes in the range of 1–100 nm [7, 8]. One of the important benefits of nanomedicine is the capability to design special NPs for detection of early osteoarthritic changes in cartilage tissue, e.g., using a liposome containing an antibody to type II collagen, which when combined with a dye emitting near-infrared light enables detection with in vivo optical imaging techniques [9]. Furthermore, NPs containing anti-inflammatory drugs and proteins (i.e., anabolic growth factors) are able to release these therapeutics in a prolonged fashion, ensuring sustained release and delivery, which is an important goal for disease therapy [10, 11]. However, the side effects of these drugs increase with higher doses. These drugs can be loaded on nanocarriers to reduce and optimize dosage and mitigate their side effects. A variety of bio-based materials such as chitosan, bovine serum albumin, hyaluronic acid (HA), and chondroitin sulfate can be used for the synthesis of NPs [12–20]. Liposomes are extensively used for drug delivery in OA due to their biodegradability, biocompatibility, and high encapsulation capacity, as well as the ability to entrap hydrophilic and lipophilic drugs [21]. This approach has been applied for intra-articular delivery of several non-steroidal anti-inflammatory drugs (NSAIDs) to prevent gastric ulceration and other side effects. Micelles are beneficial in delivery of siRNA [22]. Quantum dots [8] are effective for the recognition of MMP activity in damaged cartilage and other tissues, particularly those coated with streptavidin and conjugated with biotinylated peptide ligands [23].

The aim of this narrative review is to highlight opportunities for the application of nanotechnologies in OA diagnostics, treatment, and regenerative therapy of articular tissues. We propose that nanotechnologies may offer new opportunities and advantages for the diagnosis, prognostic indication, and treatment of osteoarticular disorders, the smart delivery of novel and conventional drugs and biological agents, and the development of biomimetic regenerative platforms for delivering gene and cell therapies to promote cartilage and bone repair.

## Osteoarthritis: From Incidence to Clinical Management

OA is the most common form of degenerative joint disease and one of the most chronic musculoskeletal diseases affecting 240 million people across the world [24–30]. In the USA alone, the cost of treatment is just over $185 billion per year. The impact of OA on society is substantial, grossly under-estimated, and increasingly a cause of concern about the ability of healthcare systems to cope with the rising socioeconomic burden.

**Fig. 1:**
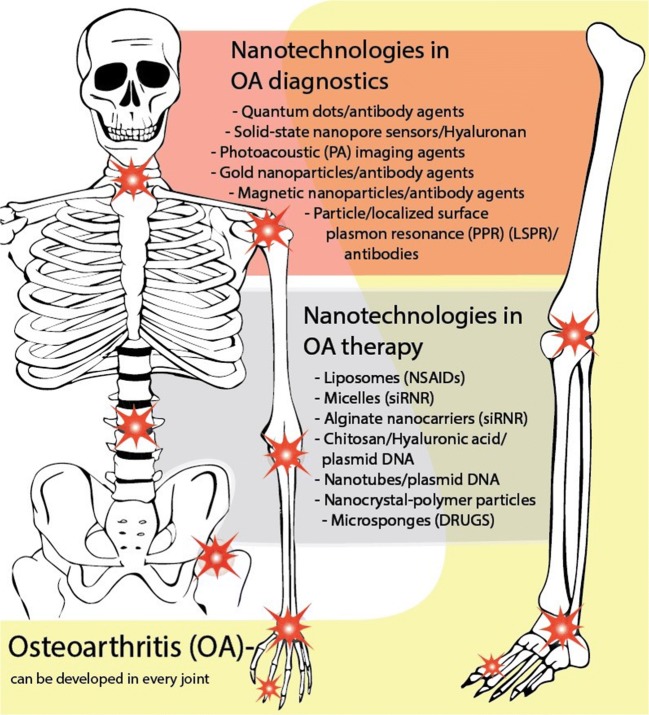
Summary of nanotechnology-based applications in osteoarthritis diagnostics and therapy

**Fig. 2:**
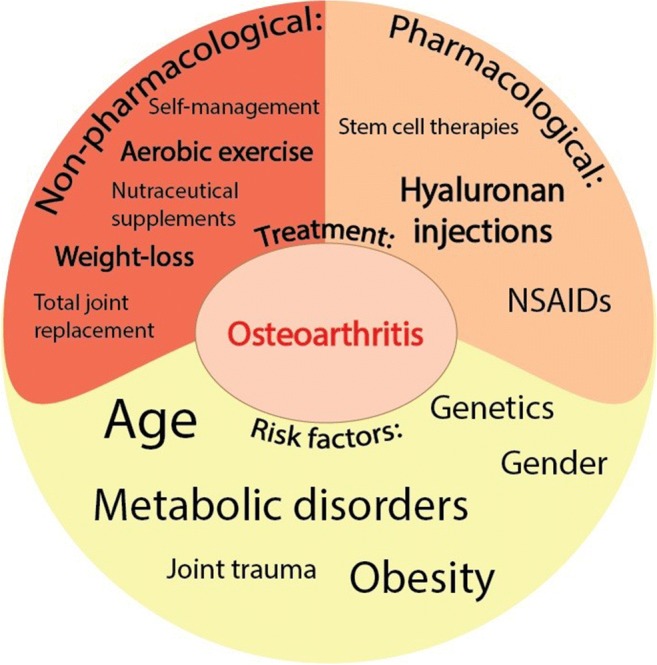
Summary of the risk factors for the development of osteoarthritis and the current pharmacological and non-pharmacological treatments for its treatment, highlighting the paucity of effective treatments and the opportunity for innovation in this area

OA ordinarily manifests in knees, hips, hands, spine, and to a lesser extent in ankles and feet (Fig. 1). The most important risk factors for the development of OA include age, overweight/obesity, joint trauma/instability, gender, genetics, and metabolic/endocrine diseases such as diabetes and crystal deposition disorders such as gout [29] (Fig. 2). Low-grade inflammation [31–33] and abnormal mechanical load [34–36] are important contributors to the onset and progression of OA [37], leading to the impaired balance between anabolic and catabolic activities in the joint [38]. Genetic factors are associated with OA, 39 to 65% for knee OA and up to 60% for hip OA [39, 40]. Since OA is an age-related disease, its incidence is higher in people between 55 and 64 years [41]. Gender is an important risk factor in the pathogenesis of OA. The prevalence, incidence, location, and severity of OA are different in men and women. Although overall the incidence rate of OA is higher in males, as compared to females [42], estimates of World Health Organization (WHO) suggest that the incidence of OA in men and women older than 60 years of age is 9.6% and 18%, respectively [43].

Although increased human life expectancy is associated with increased incidence of OA, there has been an alarming rise in the incidence of OA since the beginning of the post-industrial era. A study conducted by Wallace et al. [44] has suggested that post-industrial modernization is associated with the higher incidence of OA, such that the incidence of knee OA has been higher than early industrial and prehistoric eras. Moreover, they demonstrated that enhancements in longevity and body mass index (BMI) have not been the major reasons for the prevalence of knee OA in the USA since the mid-twentieth century.

In contrast to rheumatoid arthritis (RA), for which many treatments are currently available, there is no effective treatment for OA. A number of pharmacological and non-pharmacological therapies have been developed for the management of OA, which are largely based on symptom modification, decreasing pain, and increasing joint performance [45]. However, disease modification in OA has remained a major challenge, and the futility of currently available treatment is a source of frustration for OA patients and healthcare professionals. NSAIDs are usually administered for symptom modification. However, NSAIDs are not disease-modifying agents and, therefore, do not alter the course of disease progression. Furthermore, their long-term administration is associated with adverse side effects on the renal, gastrointestinal, and cardiovascular systems [46]. In order to remedy OA, there are currently two options in clinical use: (1) non-pharmacological treatments and (2) pharmacological treatments (Fig. 2). For non-pharmacological therapy, a number of guidelines are available along with recommendations that may be presented to the patients, including weight loss, aerobic exercise, and self-management [47–50]. Metabolic syndrome [51, 52] and immunometabolic alterations [53, 54], obesity [55], dyslipidemia [56], hyperglycemia, and insulin resistance [57] are all associated with the increased rate of OA. Therefore, physical exercise and weight loss are considered as the most effective interventions for the prevention and treatment of OA [47, 48]. With regard to pharmacological therapies that are currently in clinical use, they not only address the symptoms of the disease (i.e., pain), but also aim to impact on the progression of the disease. In line with this strategy, a variety of nutraceutical supplements such as diacerein, glucosamine, and chondroitin sulfate have been examined [58]. Biological agents that target cartilage extracellular matrix (ECM) degradation, bone remodeling, inflammation, and dysfunction of skeletal muscle, as well as adipose tissue metabolism, are considered to be potential candidates for the treatment of OA. Although these approaches seem promising, there is still no effective, approved treatment for OA, which could eliminate the necessity of surgical intervention in most of the cases. Arthroscopic debridement, allograft application, autologous chondrocyte implantation, and matrix-based autologous chondrocyte implantation are methods commonly used to repair focal and isolated cartilage lesions [59–61]. These methods allow partial restoration of mobility and help to ameliorate the symptoms of cartilage damage [62]. However, the newly formed tissue, which primarily consists of fibrocartilage, a “scar” tissue, instead of native hyaline cartilage, is very fragile and mechanically weak. Therefore, tissue engineering techniques applying three-dimensional (3D) scaffolds loaded with cells, as well as nanocompound-based drug delivery systems, seem promising approaches for the development of new therapies for cartilage lesions [63, 64]. Molecular components and biochemical signals that control differentiated chondrocyte function and promote proper cartilage structure formation, pore size, and mechanical competence should be carefully considered when developing such techniques for the regeneration of damaged cartilage tissue [65]. However, total joint replacement surgery, such as total knee or hip replacement, has been demonstrated as the most effective last-resort treatment for severe forms of OA [66, 67]. Therefore, it is vital to develop novel tools and strategies for early diagnosis and treatment of OA.

## Novel Pharmacological Treatments and Molecular Pathways

In recent years, much attention has been focused on the elucidation of the molecular pathways involved in the onset and progression of OA, and autophagy is one of the most interesting and promising areas. Research suggests that the loss of autophagy, a homeostatic mechanism, is related to the pathogenesis of OA, as reduced autophagy can be observed in cartilage in late OA consistent with increased levels of chondrocyte apoptosis [68, 69]. A large body of evidence is emerging to suggest a role for peroxisome proliferator-activated receptor gamma (PPARγ) in preserving mTOR signaling, which leads to the inhibition of autophagy [70]. On the other hand, findings have demonstrated the dual role of transforming growth factor-beta (TGF-β) signaling pathways in articular cartilage homeostasis via inhibition of terminal chondrocyte maturation and in the pathogenesis of OA by induction of pathological alterations in the subchondral bone [14, 71]. Thus, the regulation of this important signaling pathway can be considered among the potential strategies for inhibiting OA progression. Other pathways such as heparin-binding epidermal growth factor-like growth factor (HB-EGF) [72], fibroblast growth factor (FGF) [73], and hypoxia-inducible factor 1α [74] are under investigation to identify targets to prevent and treat OA.

According to various published guidelines for management of OA, the pharmacologic option is a priority after exercise, self-management, and education. Among pharmacological approaches, natural products have attracted much attention due to their valuable biological properties and low probability of side effects [75, 76]. Chondroitin, avocado/soybean unsaponifiables, curcumin, methylsulfonylmethane, willow bark extract, and pycnogenol are the most popular natural products for modulation and management of OA. However, the discussion of nutrition, dietary factors, and nutritional supplements containing natural products is beyond the scope of this review, and therefore, we would like to direct readers to several recent narrative and systematic reviews on this topic [77–81].

## Traditional and New Nanoparticle-Based Technologies for Osteoarthritis Diagnosis

### Nanoparticles for Imaging Diagnostics

Although radiography is still the first and most widely used imaging method for assessment of a patient with a suspected or known diagnosis of OA, precise measurement of articular structures is not possible by X-ray. Computed tomography (CT), which is another radiogrphic technique, can also only indirectly assess cartilage degeneration by 3D observation of joint space narrowing, which is a feature of late-stage OA [82, 83]. While it is possible to use a contrast agent in CT to directly visualize articular cartilage in 3D in vivo [84], this approach is invasive as it requires an injection of the contrast agent directly into the joint space.

More sensitive non-invasive imagining techniques, for example, ultrasound and magnetic resonance imaging (MRI), allow visualization of changes in the cartilage volume or thickness. In clinical medicine, the most commonly used non-invasive medical imaging technique to visualize the structural changes associated with functional changes in tissues is MRI [85–87]. Moreover, by developing new contrast agents, MRI can be applied to permit the more accurate visualization of structural tissue changes. For example, due to high biocompatibility and low toxicity, iron-based magnetic nanoparticles (MNPs) have been developed for clinical oncology imaging as novel biomarker-specific agents [88]. Furthermore, MNPs could also be used as multifunctional agents because it is possible to combine diagnostic and therapeutic properties into them [89]. For example, superparamagnetic iron oxide nanoparticles (SPIONs) exhibit a high degree of saturation magnetization, which is lost in the absence of magnetic field. These NPs are considered relatively less toxic than optical agents. For active targeting, SPIONs can be embedded into polymer cores and conjugated with different peptides, antibodies, or small molecules. In this way, modified SPIONs could serve as agents for the detection of OA biomarkers in synovial fluid [90].

Iron oxide NPs have also been proposed for use in apoptosis detection in transplanted stem cells in arthritic joints. A significant problem for the long-term success of the matrix-associated stem cell implants (MASI) or chondrocyte implants (MACI) is the loss of cells after implantation due to cell migration, necrosis, and apoptosis [91, 92]. Clinically, the success of implantation is usually assessed only a few weeks following the cell implantation by invasive arthroscopy and biopsy [93–95]. Therefore, early detection and visualization of cell implants is an essential aspect in the development of similar strategies for OA treatments. In vitro studies have shown that iron oxide particles directly accumulates in the cytoplasm of viable cells, while ferumoxides are dispersed in large amounts in smaller cellular fragments following apoptotis. In addition, dispersed iron oxides showed a stronger T2 signal in apoptotic cell fragments compared to iron oxide clusters in viable cells [96].

Non-iron-based NPs, for instance, gadolinium (Gd)-based contrast agent and caspase-3-sensitive nano aggregation MRI probe (C-SNAM), may also be successfully used for MACI and MASI implantation assessment. C-SNAM is a small molecular probe that can be easily delivered to MASI in cartilage defects by injection and passive diffusion. Induction-spectral plasma (ICP-MS) analysis showed significantly higher levels of Gd concentrations in apoptotic adipose-derived stem cells (ASCs) than in viable ASCs. Moreover bioluminescence imaging studies confirmed apoptosis of mitomycin C-exposed cells. In vivo studies demonstrated that strong bioluminescent signals were detected in all ASCs implants immediately after implantation. In addition, C-SNAM-exposed apoptotic cells also showed significantly stronger T1 signals on MRI than viable cells [97]. However, it should be noted that many visualization methods are still in the experimental phase and are not yet standardized sufficiently for use in classification during daily clinical practice [97]. However, it should be noted that many visualization methods are still in the experimental phase and are not yet standardized sufficiently for use in classification during daily clinical practice [98].

A novel and innovative alternative to MRI for detection of cartilage degeneration is photoacoustic imaging (PAI). PAI is a hybrid bioimaging technology that combines the benefits of ultrasound with deep penetration into tissues and optical imaging with high spatial resolution [99, 100]. Although the optical image has limited use for detection of OA, PAI can visualize neovascularity in arthritic joints, as well as provide morphological information on the degeneration of finger joints [101, 102]. However, contrast agents used currently for PAI lack the sensitivity and specificity for detecting cartilage lesions in the early stages of the disease. NP-based contrast agents such as cationic poly-L-lysine-enveloped water-soluble anionic melanin nanoparticles (PLL–MNPs), which have desirable properties of biocompatibility and low cytotoxicity, could be used to improve the sensitivity of PAI [103–105]. Research shows that PLL–MNPs can enhance PAI with significantly different accumulation in OA compared to healthy joints [106]. Interestingly, comparative analysis of results from PAI, radiography, and MRI show that PAI based on PLL–MNPs could be more useful for the detection of cartilage degeneration than standard joint examination methods. Moreover, histological results are consistent with PAI and confirmed the feasibility of PAI using PLL–MNPs to detect cartilage degeneration in early-stage disease [106].

One of the obstacles in evaluating pharmacological intervention involves the definition, identification, and quantification of early OA, as well as following up the efficacy of the applied therapies. Therefore, early detection and accurate visualization of cartilage degeneration and other synovial joint alterations are crucial for the appropriate treatment of OA.

### Nanomaterial-Based Biosensors and Biomarkers for Early Diagnosis

The current clinical diagnosis of OA is based traditionally on clinical symptoms (e.g., pain and loss of function) and radiographic criteria (e.g., joint space width), which often occur late in the disease course. An attractive and practical alternative could be the measurement of biochemical markers, which can reflect dynamic events such ECM synthesis and degradation. Biomarkers may be measured in body fluids such as synovial fluid, blood (serum or plasma), and urine [107]. The discovery of a definitive biomarker and its utilization in clinical practice could help to diagnose the disease much earlier and, importantly, to distinguish between phenotypes [108], the fast and slow progressive forms of the disease, and the erosive and non-erosive forms of OA [109, 110]. For these reasons, the main proteins of the ECM of cartilage, the metabolic products, and inflammatory mediators are widely examined. Many of these biomarkers are associated with the metabolism of collagen type II or aggrecan in cartilage or of collagen type I in subchondral bone [111–113].

Other biomarkers that could reflect an initial change of ECM structure are related to a range of non-collagenous matrix proteins, including glycoproteins and proteoglycans, as well as matrix-degrading metalloproteinases. These molecules are constituents of both cartilage and synovium, and they have a role in other metabolic pathways in the joint.

Technological methods that have been developed for OA biomarker detection include enzyme-linked immunosorbent assay (ELISA), real-time polymerase chain reaction (RT-PCR), quartz crystal microbalance, mass spectrometry, and electrochemical methods. Some of these methods offer high sensitivity or selectivity; however, they have essential disadvantages not only because they are time-consuming and costly, but also they have reduced precision. For this reason, methods with high sensitivity and selectivity, as well as small sample volume requirements, are under investigation for their potential to detect OA in its early stages. These include nanoparticle-based devices or label-free and real-time biosensors for specific detection of OA biomarkers such as glycosaminoglycans (GAGs) released from degrading cartilage, HA, cytokines, free radicals (NO), and proteinases.

Loss of aggregating proteoglycans and constituent GAGs, which are essential contributors to the structure and biomechanical properties of articular cartilage, usually occurs before significant morphological changes [114].

HA is a linear polysaccharide that is commonly found in synovial fluid, as well as in the skin and other tissues and organs. Its molecular mass ranges from 10^5^ to 10^7^ Da. This corresponds to 250–25,000 units of disaccharides [115]. High-molecular-weight HA (> 1000 kDa) displays immunosuppressive properties [116]. On the other hand, low-molecular-weight HA (typically < 500 kDa) has pro-inflammatory effects and can stimulate the production and secretion of inflammatory cytokines [117]. Therefore, the size distribution and abundance of HA is considered a promising biological indicator of pathophysiology and could be assessed as a target for disease-specific diagnostics [98, 118, 119].

Currently, to determine the molecular mass of HA or similar molecules, ELISA, size exclusion chromatography (SEC), and multi-angle laser light scattering (MALLS) methods can be used. However, they all have significant limitations, such as limited ability to distinguish molecular weights of molecules (ELISA), limited practical constraints on the number of fractions and samples that can be tested (SEC) or limited accuracy, and are relatively insensitive for low molecular weight fragments (MALLS). For these reasons, a label-free solid-state nanopore sensor was developed using a nanometer-scale aperture formed in a thin membrane as the only fluid connection between two reservoirs of an electrolyte solution [120]. After analyzing the translocation properties of the molecules, the molecular weight of HA or other molecules could be determined on a per molecule basis, and finally, overall size distribution is obtained from only a few hundred events. For example, SS-nanopore-based detection method has been tested in the studies of an equine model of OA [120]. Results confirmed that analysis of translocation properties using this method is sufficient for determining the size distribution and physiological concentration of HA in biological fluids and could be a good alternative for assessment of another OA significant molecular biomarkers.

Proteolytic enzymes, such as MMP-3, MMP-13, or ADAMTS, could be other essential biomarkers for the early diagnosis of OA. However, for determination of the activity of MMPs and ADAMTS, anti-neoepitope antibodies that recognize relatively large substrates are usually used. In some cases, new anti-neoepitope antibodies lack specificity for a particular enzyme because other proteases can also produce similar neo-peptides [121, 122]. Therefore, the use of non-antibody-based methods could be more appropriate for the detection of ADAMTS or other MMPs than current techniques. For instance, a new fluorescent probe (probe ADAMTS-4-D-Au) based on AuNPs to determine the activity of ADAMTS-4 have been developed. The method employs fluorescein isothiocyanate (FITC) linked to the N-terminal of the ADAMTS-4 specific peptide DVQEFRGVTAVIR (Asp-Val-Gln-Glu-Phe-Arg-Gly-Val-Thr-Ala-Val-Ile-Arg) with the FITC-peptide conjugated to AuNPs with a diameter of 7 nm through cysteine by a gold-thiol bond [123]. This probe is stable under physiological conditions, and fluorescence intensity is proportional to the concentration of active ADAMTS-4. When this ADAMTS-4-D-Au probe was used to determine ADAMTS-4 activity in human synovial fluid, the strongest fluorescence signals were detected in patients with acute joint injury and patients with late-stage OA [123].

Moreover, the high activity of ADAMTS-4 was consistent with the results obtained from the arthroscopy analysis, while the MRI results were different. For instance, for a patient from the group with acute joint damage, arthroscopy revealed second-degree cartilage damage; however, T1-weighted and T2-weighted MRI did not specify any difference related to cartilage damage, while fluorescence intensity in this patient was comparatively high. This probe can be used to identify ADAMTS as a potential biomarker associated with cartilage damage at an early stage of the disease [123].

Alternative non-antibody-based method for non-invasive, real-time evaluation of OA has been developed for the monitoring of the nitric oxide (NO) release in OA chondrocytes, which overexpress the gene encoding inducible NO synthase (NOS2) and its product, NO [124, 125]. NO has been considered as a biomarker for OA [126]. The nanosensors were synthesized by encapsulating the NO-sensing molecules (4-amino-5-methylamino-2′,7′-difluorofluorescein diaminofluorescein-FM (DAF-FM)) within the biodegradable poly-(lactic-co-glycolic acid) NPs. In vitro studies have demonstrated that there is a positive correlation between the increase in the fluorescence intensity and the change in NO concentration in the chondrocytes. The efficacy of this approach was tested in the rat model of OA due to anterior cruciate ligament transection (ACLT). After ACLT surgery, the level of NO in the joint fluid increased with cartilage degeneration and was positively correlated with increased NO nanosensor fluorescence [127].

Another promising technology for OA diagnostics with improved sensitivity and analysis time is based on the chemical properties of gold nanoparticles (AuNPs), which are being extensively studied for the development of new multimodal contrast elements or biosensors. Gold nanoparticle biosensing involves the interaction between a target biomarker molecule and a AuNP crosslinker or a AuNP-containing antibody [128].

For biosensor applications, AuNPs are attractive for their chemical stability and convenient spectral window in the visible range. The AuNP-based biosensors are designed to be selective for the detection of various biomolecules, including small molecules, peptides, and nucleic acids.

Particle plasmon resonance (PPR) or localized surface plasmon resonance (LSPR) methods are used widely for the determination of chemical and biochemical species, because of the sensitivity of their electron-rich surfaces to the surrounding environment [129–131]. For example, a fiber-optic particle plasmon resonance (FOPPR)-sensing platform, based on AuNP-modified optical fiber for the detection of the OA related pro-inflammatory cytokine interleukin (IL)-1β in synovial fluid samples, has been developed [132]. In this FOPPR sensing platform, the molecular binding of IL-1β on the AuNP-conjugated anti-IL-1β transduces a local increase in the refractive index of the medium surrounding the AuNP, enhancing the plasmon absorbance of the AuNP. Linear regression analysis showed a good correlation coefficient for both ELISA and modified FOPPR detection methods. However, using a FOPPR sensor, the analysis time for detecting IL-1β in synovial fluid was significantly shorter than with ELISA, thereby reducing the chance of potential experimental errors. Similar results were obtained using FOPPR sensor for detection of TNF-α and MMP-3, achieving an excellent refractive index resolution (5.18 × 10–7 RIU) with limits of detection as low as 0.48 pM and 1.56 pM for TNF-α and MMP-3, respectively. This suggests that the label-free and real-time detection capabilities of the FOPPR sensor for protein analysis could be an excellent alternative to immunoassay [133].

## Target Nanocarriers for OA Therapies

### Nanoparticles for Drug Delivery

Studies have shown beneficial properties of NPs in systems for targeted drug delivery and sustained release, making them attractive tools for OA treatment (Table 1). Investigation of self-assembled thermoresponsive nanostructures of HA conjugates [156], administered through subcutaneous and intra-articular injections, has shown beneficial features, including good biocompatibility, sustained drug release, cartilage protection, reduction of inflammatory cytokines such as IL-1β and TNF-α, and maintenance of epiphysis thickness. Nanocrystal-polymer particles have been designed as potential drug delivery carriers for OA treatment [154]. Nanocrystals (NPPs) of kartogenin (KGN), prepared by wet milling and loaded subsequently with polymer microparticles (320 nm), demonstrated high drug loading (31.5% w/w) and prolonged drug release (62% over 3 months). In vitro experiments showed that KGN-NPPs do not change the mitochondrial activity of cultured human OA synoviocytes. In a murine mechanistic OA model in vivo, the KGN-NPPs show higher bioactivity compared to KGN in solution and sustained intra-articular persistence without any irritation [154]. On the other hand, p38 MAPK inhibitor (PH-797804)-loaded nanostructures (PH-NPPs) are promising for the management of OA [155]. The PH nanocrystals prepared by wet milling and embedded into fluorescent particles were stabilized with D-ɑ-tocopheryl polyethylene glycol 1000 succinate. The PH-NPPs showed beneficial properties in terms of good diameter (14.2 μm), high drug loading (31.5%), prolonged drug release (20% PH release within 3 months), and biocompatibility; PH-NPPs resided in the joint and adjacent tissues for 2 months, associated with decreased levels of inflammatory factors, IL-1β, IL-6, and IL-17, and attenuated inflammation and joint damage.

**Table 1:** Drug delivery systems for OA treatment

Nanocarrier	Drug	Cell line/Animal model	Major outcomes	Refs
PLGA nanoparticle	WYRGRL peptide	Model of OA	Biodegradable and specific binding to the cartilage tissue	[134]
PEG poly (NIDAM) NPs	KAFAK	OA model	Effective drug delivery and inhibiting the pro-inflammatory IL-6 expression	[135]
Niosome	Date seed oil	Cg-induced paw edema	Good stability, nano-size range, and great anti-inflammatory activity	[136]
Bisphosphate nanoparticle	Clodronate	Circulating progenitor cells (CPCs) OA model	Upregulation of SOX9 gene expression upon treatment, decreased osteoarticular pain, and improved mental and physical performance	[137]
PVCL-co-acrylic hydrogels	Sodium diclofenac	–	Sustained permeation through an artificial skin membrane and high drug delivery	[138]
Lipid nanoparticle	Ibuprofen	Male SKH-1 hairless mice	High entrapment efficiency (95.51%), high permeation, and potential anti-inflammatory activity	[139]
PLGA nanoparticle	IL-1 receptor antagonist (IL-1Ra)	NF-KB inducible reporter cell line	Tunable size (300-700 nm), cytocompatible, good stability, and efficient inhibition of IL-1β signaling	[140]
AuNPs	Chondroitin sulfate	Primary goat chondrocytes	High increase in GAG and collagen production, stimulating chondrocyte proliferation, and enhancing extracellular matrix production	[141]
Chitosan NPs	Berberine	Rat knee OA model	Spherical shape, good stability, ideal releasing profile, increased retention time in synovial fluid and high anti-apoptotic activity	[20]
Solid lipid NPs	Aceclofenac	Albino rat	Good particle size (143.4–154.2 nm), prolonged drug release, high uptake, and great anti-inflammatory activity	[12]
Lipid NPs	Diacerein	Rat model of OA	Good particle size (396 nm), sustained release, high delivery, and improved histopathology analysis	[142]
Lipid NPs	Diacerein	Rat	Good particle size (270 nm) and zeta potential (− 13.78 to − 19.66 mV), high entrapment efficiency (88.1%), sustained drug release, and decreased side effects of diacerein	[143]
NPs-in-microspheres	Brucine	Rats	High biocompatibility, prolonged drug release, high residence in articular cavity, and improved retention	[14]
Polymeric NPs	KAFAK	THP-1 cells Cartilage plugs	Decreased pro-inflammatory cytokine and selective targeting	[144]
Polymeric NPs	Curcumin	Human primary chondrocytes	Inhibiting mRNA expression of pro-inflammatory mediators (IL-1β; TNF-α; MMPs 1, 3, and 13), decreasing OA disease progression, reducing proteoglycan loss, and decreasing synovitis	[145]
PEGylated NPs	KAFAK	Chondrocytes	Efficient targeted delivery and decreasing inflammatory reaction	[146]
Polymeric NPs	IL-1Ra protein	Synoviocytes Rat stifle joint	Good particle size (300 nm), maintaining bioactivity, specifically targeting synoviocytes, increased retention time, and decreasing inflammatory factors	[19]
Bipolymeric NPs	Dextran FITC	Healthy rat knees	No decrease in proteoglycans biosynthesis and induction no inflammatory response	[147]
PLGA NPs	Dexamethasone	Synovium and articular damage	Excellent biocompatibility, internalization via phagocyte process and stimulation of inflammation	[148]
Polymeric NPs	Curcumin	Rat model of OA	Enhancing cellularity and matrix and high biocompatibility	[149]
Coiled-coil protein	BMS493	Human articular chondrocytes	Reducing mRNA levels of MMP-13 and IL-1β	[150]
Ginger extract nanoparticle	–	Patients with knee OA	Improving knee joint pain, symptoms, daily activities, and quality of life	[151]
Solid lipid NPs	Diacerein	–	High encapsulation of diacerein, prolonged release behavior, increase in diacerein payload and thermoresponsive drug delivery	[152]
Amine terminal polyamidoamine (PAMAM) dendrimres	Insulin-like growth factor 1 (IGF-1)	Rat OA	Promoting pharmacokinetics and potential of disease-modifying OA drugs	[153]
Nanocrystals-polymer particles	–	Human OA synoviocytes and murine mechanistic OA model	Lack of effect on mitochondrial activity, exerting protective effect on the cartilage and epiphysis of the medial tibia, and significant reduction in VEGF and Adamts5 expression	[154]
Nanocrystal-polymer particles	P38a/b MAPK inhibitor PH-797804	OA model and human OA synoviocytes	Lack of toxicity against human OA synoviocytes, decreasing inflammation and joint destruction and also excellent retention and function at the target site	[155]
Self-assembled thermoresponsive nanostructures of hyaluronic acid		OA mouse model	High biocompatibility and significant sustained residence time at the injection site, reduction of inflammatory cytokines and efficacy in delivery of peptides, proteins or small molecules	[156]
Poly (ester-amide) particle	Celecoxib	Ovine model	High biocompatibility, no toxic effect at the injection site, and great diffusion into neighbor tissues	[157]
HA-PLGA particles	–	RAW264.7 macrophage cells and Wistar rats	Great safety and high anti-inflammatory effect	[158]

Furthermore, Avidin nanocarriers are appropriate for intra-cartilage delivery of dexamethasone (DEX) [159]. Using DEX-Avidin conjugates, prepared using fast (ester) and slow, pH-sensitive release (hydrazine) linkers, the DEX was rapidly released from the conjugates, resulting in high bioactivity. In cartilage explants in vitro, a single dose of Avidin-DEX significantly inhibits the cytokine-induced loss of sulfated-glycosaminoglycan (sGAG), as well as decreasing and even suppressing IL-1α-induced cell death and enhancing sGAG synthesis levels [159]. As a novel strategy in the treatment of OA, KGN-conjugated chitosan nano-microparticles can promote cartilage regeneration [15]. These carriers demonstrate excellent properties in terms of prolonged release (7 weeks), strong stimulatory effects on the expression of chondrogenic markers in vitro, long retention time in the knee joint after intra-articular injection, and inhibitory effects on cartilage degeneration in vivo. KGN-conjugated polyurethane NPs (PN-KGN) have demonstrated great potential for OA treatment [160]. These spherical nanocarriers with a mean size of 25 nm release KGN in a sustained behavior. Notably, the nanocarriers are biocompatible, having no cytotoxicity or pro-inflammatory impact on cells. Intra-articular administration of these NPs decreases cartilage degeneration remarkably, resulting in inhibition of OA development [160]. Polypeptide nanogels (PNGs) with encapsulated methotrexate (MTX) were investigated for improving collagen-induced arthritis [161]. PNG-MTX showed glutathione (GSH)-triggered release behavior similar to that of MTX alone. Moreover, PNG-MTX had high internalization and toxicity against activated macrophages. PNG-MTX treatment in vivo remarkably decreases arthritic scores and diminishes paw thickness, suggesting potential anti-inflammatory activity. Histopathological analysis of the PNG-MTX group revealed decreased numbers of inflammatory cells, normalization of cartilage morphology, joint space widening, and decreased roughening of the articular surface [161].

Silk fibroin microparticles (SFMs) have been designed for intra-articular drug delivery [162]. SFMs are spherical in shape with particle size in the range of 598 nm to 21.5 μm and show prolonged release and retention in the joint. Furthermore, curcumin-loaded solid lipid nanoparticles (Cur-SLNs) exhibit high efficacy for alleviation of adjuvant-induced arthritis [163]. Cur-SLNs (10 and 30 mg/kg) effectively decreased joint hyperalgesia, joint stiffness, and paw volume, as well as improving the mobility score, reducing blood leukocyte count, and decreasing oxidative stress, TNF-α, and C-reactive protein. HA-chitosan nanoparticles (HA-CNPs) are also appropriate for delivery of curcuminoid in knee OA treatment because of their high drug loading capacity (38.44%) and prolonged drug release behavior [164]. In a knee OA model, using the Hulth-Telhag surgical procedure and co-treatment with IL-1β and TNF-α, the administration of curcuminoid-loaded HA-CNPs significantly reduced the Outerbridge and Mankin pathological scores to close to normal until the fourth week. The curcuminoid-loaded HA-CNPs also significantly suppressed NF-kB signaling and expression of the metalloproteinases MMP-1 and MMP-13, whereas they upregulated the collagen II expression in chondrocytes in vitro [164].

### Nanoparticles for Gene Delivery and Gene Therapy

The challenges of chemoresistance to drugs and associated side effects have opened up new opportunities in the field of gene therapy [165], offering novel perspectives for the design of biocompatible, biomimetic, and efficient gene carriers. Non-viral gene delivery using nanocarriers and scaffolds is a promising approach for disease therapy [166–168] (Table 2). For instance, carbon dots in complex with the gene encoding TNF-α have been designed to facilitate stem cell-based therapy of cartilage defects [179]. In a rabbit OA model, chitosan-graft-polyethylenimine (PEI)-DNA NPs [170], which demonstrate high transfection efficiency and good biocompatibility confirmed by cell viability assay, is able to deliver plasmid-DNA (pDNA) into the nuclei of chondrocytes and synoviocytes. Image-guiding, photothermal-triggered hemoglobin (Hb)-based NPs can absorb near-infrared light at 650 nm (0.5 W cm^−2^) and convert it into heat. The Notch1-siRNA-loaded NPs act by suppressing macrophage inflammation, suggesting that this is potential biocompatible nano-platform for clinical OA therapy [179].

**Table 2:** Gene deliveries for OA treatment

Nanocarrier	Gene	Cell line/Animal model	Major outcomes	Refs
Iron oxide NPs	SiRNA against IL-2/-15 receptor β chain	Arthritic rats	Biocompatible, improved siRNA stability, high uptake by macrophages, and great anti-inflammatory effect	[169]
Chitosan NPs	DNA (plasmid)	Chondrocytes and synoviocytes	High transfection efficiency, great biocompatibility, and delivery of pDNA into the nucleus of chondrocytes and synoviocytes	[170]
Calcium phosphate/liposome NPs	NF-kB targeted DNA	Arthritic rats	Inhibiting the progression of OA by targeting macrophages and decreasing pro-inflammatory cytokines by inhibiting NF-kB signaling pathway	[171]
Hyaluronic acid/chitosan NPs	Plasmid-DNA	Chondrocytes	High transfection efficiency and increasing the viability of chondrocytes	[172]
Chitosan NPs	IL-1Ra or IL-10 genes	Osteoarthritic rabbits	Improving histologic lesions and decreasing inflammation	[173]
Chitosan-HA NPs	IL-1Ra	Synoviocytes	Sustained pDNA release, high biocompatibility, and great anti-inflammatory effect	[174]
Nanohydroxyapatite (nHA)	TGF-β3 and BMP2	MSCs	Directing MSCs fate for articular cartilage and endochondral bone tissue engineering	[175]
Polymeric NPs	Anti-Hif-2α siRNA	Arthritic mice	Downregulation of Hif-2α, MMP-12 and -9, ADAMTS-4, VEGF, collagen type X and NF-kB, promoting local concentration, increasing retention time, decreasing IL-1β and attenuation of synovium inflammation	[176]
HA/chitosan NPs	Cytokine response modifier A	Rat knee osteoarthritis model	Effective entrapment of plasmid-DNA, sustained release over 3 weeks, inhibiting cartilage damage, synovial inflammation, and loss of type II collagen and downregulation of IL-1β and MMP-3 and MMP-13	[177]
Bioconjugated carbon dots with succinimidyl-4-(N-maleimidomethyl) cyclohexane-1-carboxylate (SMCC)	Silenced TNF-α (siTnfα)	MSCs	MSCs chondrogenesis enhancement by inflammation suppression	[178]
NO-hemoglobin@PLGA-PEG NPs	Notch1-siRNA	Macrophage	Suppressing macrophage inflammation	[179]

HA-chitosan-NPs [172] transfect chondrocytes with high efficiency and maintain cell viability at more than 90% [172]. Chitosan NPs carrying the gene encoding IL-1Ra exhibit high efficacy for gene delivery [173], and when injected into the knee joints of rabbits with OA, increase the levels of IL-1Ra in knee joint synovial fluid. In contrast, no IL-1Ra can be detected in the chitosan-IL-10-injected group. In addition, decreased severity of histologic cartilage lesions was detected in the group treated with chitosan-IL-1Ra [173]. Chitosan-HA-NPs carrying pDNA encoding IL-1Ra exhibit advantageous effects in alleviation of inflammation in synoviocytes, where they enhance IL-1Ra gene expression and decrease the mRNA and protein levels of MMP-3, MMP-13, cyclooxygenase-2 (COX-2), IL-1β, and iNOS [174]. These nanocarriers have zeta potential and particle size of + 28 mV and 144.9 nm, respectively, and effectively protect pDNA. The pDNA exhibits a prolonged release pattern of up to 15 days, and the biocompatibility of these nanocarriers can be confirmed by cytotoxicity assay [174].

As described above, alginate can be easily used not only as a scaffold for cell culture, but also as a nanocarrier for delivering genes to cells. Gene-activated alginate hydrogels capable of non-viral gene delivery via nanohydroxyapatite (nHP) have been developed to control differentiation potential of mesenchymal stem cells (MSCs) for either cartilage or endochondral bone tissue engineering [175]. For this purpose, MSCs and nHP complexed with DNA, encoding TGF-β3, BMP-2, or a combination of both (TGF-β3/BMP2), were encapsulated into alginate hydrogels [175].

Nanotubes comprise another interesting tool applied in cartilage tissue engineering, since they can be delivered directly to the cytoplasm in the cell. Among the different types of nanotubes, polyethylene glycol (PEG) chain-modified single-walled carbon nanotubes (PEG-SWCNTs) were able to efficiently enter the cartilage ECM, translocate into the cytoplasm of chondrocytes, and deliver gene inhibitors without affecting cartilage homeostasis [180]. This approach can control molecular functions of cells, which is an important option for improving cellular differentiation capability.

## Nanomaterials-Based Scaffolds for Cartilage Regeneration

Scaffolds are a key element that enables tissue regeneration. The requirements to create a scaffold include biocompatibility; complex structure (hierarchy and porosity); mechanical strength and flexibility; promotion of cell attachment, migration, and proliferation; and minimal inflammatory and immunological response. In addition to hierarchical structure and porosity of scaffolds, nano-topography is an important cue for cell adhesion, proliferation, and differentiation [181]. The effect of nano-topography on chondrogenic differentiation of MSCs was investigated via thermal nanoimprinting of PCL film. The results indicated that nano-topographical patterns affect the morphology, cytoskeletal structure, cell aggregation, and differentiation of MSCs, resulting in specific functional outcomes. Furthermore, nanopatterned films enhance chondrogenesis of MSCs and facilitate hyaline cartilage formation compared with smooth films [182].

To incorporate nano-topography into free standing scaffolds, electro-spun nanofiber-based scaffolds have been fabricated using different polymers such as poly(ε-caprolactone) (PCL), polyethersulfone (PES), and poly (lactic-co-glycolic acid (PLGA) and evaluated for chondrocyte differentiation [183–188]. A common finding among these in vitro studies is that nanofiber-based polymer scaffolds enhance chondrogenic differentiation of MSCs. Electro-spun nanofibers have been used in the development of composites with chitosan [189] and collagen [190]. While further studies are needed to evaluate the impact of chitosan in nanofibers-based scaffolds [189], collagen-poly (vinyl alcohol) nanofiber-based scaffolds in a rabbit OA model demonstrates effective regeneration of injured joints [190].

In addition to electro-spun nanofibers, hybrid peptide nanofiber-HA membrane scaffolds have been developed [191]. This scaffold preserves cartilage morphology, reduces osteophyte formation, and maintains cartilage-specific matrix proteins in OA models in vivo [191]. Self-assembled peptide (SAP) nanofibers coupled with the neuropeptide substance P (SP) [192] has been investigated as an injectable conjugate containing different concentrations of SP and applied in a rat OA model. The results showed that SP-SAP nanofibers can promote chondrogenic differentiation and delay the progression of OA. Following similar strategies, injectable hydrogel scaffolds containing chondroitin sulfate nanoparticles (ChS-NPs) and nanohydroxyapatite (nHA) were developed for osteochondral regeneration and evaluated using a rabbit model [193]. These scaffolds enhanced hyaline cartilage regeneration with subchondral bone formation and lateral host-tissue integration.

Although polymer-based scaffolds have demonstrate good biological compatibility, they quite often lack other important properties such as mechanical strength, failing to deliver the proper cues to promote functional tissue regeneration. To overcome the challenges encountered when using polymer-based scaffolds, researchers have investigated the use of nanomaterials beyond the traditional biomaterials to create nanocomposite scaffolds capable of stimulating cell attachment, growth, and tissue regeneration. Among the available nanomaterials, carbon nanotubes (CNTs) have attracted attention due to their outstanding electrical and mechanical properties, as well as their versatility in assembly of different structures. In the field of tissue engineering in particular, CNTs present nanostructural dimensions in the scale of proteins found in the ECM, enhancing the potential to influence cell attachment, proliferation, and differentiation [194]. Primary chondrocytes proliferate and align on 2D pristine CNT sheets and express high levels of ECM proteins when cultured in 3D pristine CNT textile made out of aligned CNT fibers [195]. CNTs have been also used to reinforce polymer-based scaffolds [194]. Functionalized single-wall CNTs (SWCNTs) can strengthen the mechanical properties of agarose hydrogels, while providing the optimal structure needed to maintain cellular viability and promote cartilaginous growth [196]. Nanocomposite films for chondrocyte growth have also been created using highly dispersed CNTs in polycarbonate urethane (PCU) [197], exploiting the possibility to provide electrical stimulation to cells via the conductivity property of CNTs. As a result, chondrocyte attachment and long-term cell densities can be enhanced by more than 50% (without electrical stimulation) and 200% (with electrical stimulation) on CNT-PCU composites compared to pristine PCU.

## Conclusions and Future Opportunities

Increasingly rapid development of nanotechnologies has offered a wide variety of novel approaches and platforms for both diagnosis and regenerative treatment in OA. Nanotubes, magnetic NPs, and other nanotechnology-based drug and gene delivery systems provide important targeting platforms for the development of OA therapeutic strategies. However, due to the complexity of molecular and cellular alterations in cartilage tissue in OA, nanocomposites are also currently under scrutiny as potential tools for efficiently building cartilage matrix for repair strategies. Many of the current cell-based therapies for OA are relatively simple injections of MSCs, primary chondrocytes, transduced chondrocytes, or mixtures of allogeneic primary chondrocytes and protein production platforms. One relevant example is the Kolon TissueGene cell-based therapy “TissueGene-C (TG-C).” This new and revolutionary cell-based therapy employs GP2–293 cells, a HEK 293-based retroviral packaging cell line used for large-scale growth factor production, in this case transforming growth factor-β1 (TGF-β1). This product concept has the capacity to over-produce TGF-β1 in sufficiently high quantities for supporting cellular therapy and regenerative applications, but the cells are simply injected into the joint, without any supporting gel or matrix. In the future, cell-based therapies will benefit from nanotechnology-based delivery 3D platforms and matrices that can better support the cell-based therapy. Transduced GP2–293 cells in TG-C may be transformed cells, but since they have been irradiated, they have lost their capacity for proliferation and cannot differentiate. After these cells carry out their TGF-β1 production duties, they will die and their remains will be cleared by joint resident inflammatory macrophages through the process of phagocytosis. Therefore, delivering cell-based therapies in a matrix of nanomaterials will enhance their survival and promote macrophage access to the dead cells, allowing the immune system to clear the debris. There is huge potential in this area for new innovations that can promote sustained delivery of chemical and biological drugs and the stabilization of cells for cell-based therapy.

## References

[1] Ahmadi Z, Mohammadinejad R, Ashrafizadeh M (2019). Drug delivery systems for resveratrol, a non-flavonoid polyphenol: emerging evidence in last decades. J Drug Deliv Sci Technol.

[2] Nadimi AE, Ebrahimipour SY, Afshar EG, Falahati-Pour SK, Ahmadi Z, Mohammadinejad R (2018). Nano-scale drug delivery systems for antiarrhythmic agents. Eur J Med Chem.

[3] Eichaker LR, Cho H, Duvall CL, Werfel TA, Hasty KA (2014). Future nanomedicine for the diagnosis and treatment of osteoarthritis. Nanomedicine..

[4] Holyoak DT, Tian YF, van der Meulen MC, Singh A (2016). Osteoarthritis: pathology, mouse models, and nanoparticle injectable systems for targeted treatment. Ann Biomed Eng.

[5] Roy K, Kanwar RK, Kanwar JR (2015). Molecular targets in arthritis and recent trends in nanotherapy. Int J Nanomedicine.

[6] Ouyang Z, Tan T, Liu C, Duan J, Wang W, Guo X (2019). Targeted delivery of hesperetin to cartilage attenuates osteoarthritis by bimodal imaging with Gd2 (CO3) 3@ PDA nanoparticles via TLR-2/NF-κB/Akt signaling. Biomaterials..

[7] Ajdary M, Moosavi M, Rahmati M, Falahati M, Mahboubi M, Mandegary A (2018). Health concerns of various nanoparticles: a review of their in vitro and in vivo toxicity. Nanomaterials..

[8] Mohammadinejad R, Moosavi MA, Tavakol S, Vardar DÖ, Hosseini A, Rahmati M (2019). Necrotic, apoptotic and autophagic cell fates triggered by nanoparticles. Autophagy..

[9] Noyori K, Koshino T, Takagi T, Okamoto R, Jasin H (1994). Binding characteristics of antitype II collagen antibody to the surface of diseased human cartilage as a probe for tissue damage. J Rheumatol.

[10] Maudens P, Jordan O, Allémann E (2018). Recent advances in intra-articular drug delivery systems for osteoarthritis therapy. Drug Discov Today.

[11] Brown S, Kumar S, Sharma B (2019). Intra-articular targeting of nanomaterials for the treatment of osteoarthritis. Acta Biomater.

[12] Bishnoi M, Jain A, Hurkat P, Jain SK (2014). Aceclofenac-loaded chondroitin sulfate conjugated SLNs for effective management of osteoarthritis. J Drug Target.

[13] Chen Z, Chen J, Wu L, Li W, Chen J, Cheng H (2013). Hyaluronic acid-coated bovine serum albumin nanoparticles loaded with brucine as selective nanovectors for intra-articular injection. Int J Nanomedicine.

[14] Shen J, Li S, Chen D (2014). TGF-β signaling and the development of osteoarthritis. Bone Research.

[15] Kang ML, Ko J-Y, Kim JE, Im G-I (2014). Intra-articular delivery of kartogenin-conjugated chitosan nano/microparticles for cartilage regeneration. Biomaterials..

[16] Morgen M, Tung D, Boras B, Miller W, Malfait A-M, Tortorella M (2013). Nanoparticles for improved local retention after intra-articular injection into the knee joint. Pharm Res.

[17] Ryan SM, McMorrow J, Umerska A, Patel HB, Kornerup KN, Tajber L (2013). An intra-articular salmon calcitonin-based nanocomplex reduces experimental inflammatory arthritis. J Control Release.

[18] Singh A, Agarwal R, Diaz-Ruiz CA, Willett NJ, Wang P, Lee LA (2014). Nanoengineered particles for enhanced intra-articular retention and delivery of proteins. Adv Healthcare Mater.

[19] Whitmire RE, Wilson DS, Singh A, Levenston ME, Murthy N, García AJ (2012). Self-assembling nanoparticles for intra-articular delivery of anti-inflammatory proteins. Biomaterials..

[20] Zhou Y, Liu S-Q, Peng H, Yu L, He B, Zhao Q (2015). In vivo anti-apoptosis activity of novel berberine-loaded chitosan nanoparticles effectively ameliorates osteoarthritis. Int Immunopharmacol.

[21] Patil YP, Jadhav S (2014). Novel methods for liposome preparation. Chem Phys Lipids.

[22] Li H, Yu SS, Miteva M, Nelson CE, Werfel T, Giorgio TD (2013). Matrix metalloproteinase responsive, proximity-activated polymeric nanoparticles for siRNA delivery. Adv Funct Mater.

[23] Knapinska A, Fields GB (2012). Chemical biology for understanding matrix metalloproteinase function. Chembiochem..

[24] Hosnijeh FS, Bierma-Zeinstra SM, Bay-Jensen AC (2018). Osteoarthritis year in review 2018: biomarkers (biochemical markers). Osteoarthritis Cartilage.

[25] DeFrate LE, Kim-Wang SY, Englander ZA, McNulty AL (2018). Osteoarthritis year in review 2018: mechanics. Osteoarthr Cartil.

[26] Zhai G (2019). Alteration of metabolic pathways in osteoarthritis. Metabolites..

[27] Fathollahi A, Aslani S, Jamshidi A, Mahmoudi M (2019). Epigenetics in osteoarthritis: novel spotlight. J Cell Physiol.

[28] Tonge D, Pearson M, Jones S (2014). The hallmarks of osteoarthritis and the potential to develop personalized disease-modifying pharmacological therapeutics. Osteoarthr Cartil.

[29] Bortoluzzi A, Furini F, Scirè CA (2018). Osteoarthritis and its management-epidemiology, nutritional aspects and environmental factors. Autoimmun Rev.

[30] Jones IA, Togashi R, Wilson ML, Heckmann N, Vangsness CT (2018). Intra-articular treatment options for knee osteoarthritis. Nat Rev Rheumatol.

[31] Scanzello CR (2017). Role of low-grade inflammation in osteoarthritis. Curr Opin Rheumatol.

[32] Robinson WH, Lepus CM, Wang Q, Raghu H, Mao R, Lindstrom TM (2016). Low-grade inflammation as a key mediator of the pathogenesis of osteoarthritis. Nat Rev Rheumatol.

[33] van den Bosch MH (2019). Inflammation in osteoarthritis: is it time to dampen the alarm (in) in this debilitating disease?. Clin Exp Immunol.

[34] Cohen NP, Foster RJ, Mow VC (1998). Composition and dynamics of articular cartilage: structure, function, and maintaining healthy state. J Orthop Sports Phys Ther.

[35] Andriacchi TP, Mündermann A (2006). The role of ambulatory mechanics in the initiation and progression of knee osteoarthritis. Curr Opin Rheumatol.

[36] Vincent TL, Wann AK (2018). Mechanoadaptation: articular cartilage through thick and thin. J Physiol.

[37] Griffin TM, Guilak F (2005). The role of mechanical loading in the onset and progression of osteoarthritis. Exerc Sport Sci Rev.

[38] de Andrés M, Takahashi A, Hashimoto K, Imagawa K, Oreffo RJO (2016). Cartilage. Elucidation of the epigenetic mechanisms underlying anabolic and catabolic gene regulation in osteoarthritis. Osteoarthr Cartil.

[39] Valdes AM, Spector TD (2011). Genetic epidemiology of hip and knee osteoarthritis. Nat Rev Rheumatol.

[40] Van Meurs J (2017). Osteoarthritis year in review 2016: genetics, genomics and epigenetics. Osteoarthr Cartil.

[41] Loeser RF, Collins JA, Diekman BO (2016). Ageing and the pathogenesis of osteoarthritis. Nat Rev Rheumatol.

[42] Srikanth VK, Fryer JL, Zhai G, Winzenberg TM, Hosmer D, Jones G (2005). A meta-analysis of sex differences prevalence, incidence and severity of osteoarthritis. Osteoarthr Cartil.

[43] Woolf AD, Pfleger B (2003). Burden of major musculoskeletal conditions. Bull World Health Organ.

[44] Wallace IJ, Worthington S, Felson DT, Jurmain RD, Wren KT, Maijanen H (2017). Knee osteoarthritis has doubled in prevalence since the mid-20th century. Proc Natl Acad Sci.

[45] Peat G, Thomas E, Handy J, Wood L, Dziedzic K, Myers H (2004). The Knee Clinical Assessment Study–CAS (K). A prospective study of knee pain and knee osteoarthritis in the general population. BMC Musculoskelet Disord.

[46] Hunter DJ (2011). Pharmacologic therapy for osteoarthritis—the era of disease modification. Nat Rev Rheumatol.

[47] Fernandes L, Hagen KB, Bijlsma JW, Andreassen O, Christensen P, Conaghan PG (2013). EULAR recommendations for the non-pharmacological core management of hip and knee osteoarthritis. Ann Rheum Dis.

[48] Hochberg MC, Altman RD, April KT, Benkhalti M, Guyatt G, McGowan J (2012). American College of Rheumatology 2012 recommendations for the use of nonpharmacologic and pharmacologic therapies in osteoarthritis of the hand, hip, and knee. Arthritis Care Res.

[49] McAlindon TE, Bannuru RR, Sullivan M, Arden N, Berenbaum F, Bierma-Zeinstra S (2014). OARSI guidelines for the non-surgical management of knee osteoarthritis. Osteoarthr Cartil.

[50] Zhang W, Nuki G, Moskowitz R, Abramson S, Altman R, Arden N (2010). OARSI recommendations for the management of hip and knee osteoarthritis: part III: changes in evidence following systematic cumulative update of research published through January 2009. Osteoarthr Cartil.

[51] Courties A, Sellam J, Berenbaum F (2017). Metabolic syndrome-associated osteoarthritis. Curr Opin Rheumatol.

[52] Courties A, Sellam J (2016). Osteoarthritis and type 2 diabetes mellitus: what are the links?. Diabetes Res Clin Pract.

[53] Mobasheri A, Rayman MP, Gualillo O, Sellam J, Van Der Kraan P, Fearon U (2017). The role of metabolism in the pathogenesis of osteoarthritis. Nat Rev Rheumatol.

[54] Courties A, Gualillo O, Berenbaum F, Sellam J (2015). Metabolic stress-induced joint inflammation and osteoarthritis. Osteoarthr Cartil.

[55] Oliveria SA, Felson DT, Cirillo PA, Reed JI, Walker AM. Body weight, body mass index, and incident symptomatic osteoarthritis of the hand, hip, and knee. Epidemiology. 1999:161–6. 10069252

[56] Baudart P, Louati K, Marcelli C, Berenbaum F, Sellam J (2017). Association between osteoarthritis and dyslipidaemia: a systematic literature review and meta-analysis. RMD open.

[57] Louati K, Vidal C, Berenbaum F, Sellam J (2015). Association between diabetes mellitus and osteoarthritis: systematic literature review and meta-analysis. RMD open.

[58] Permuy M, Guede D, López-Peña M, Muñoz F, Caeiro J-R, González-Cantalapiedra A (2015). Comparison of various SYSADOA for the osteoarthritis treatment: an experimental study in rabbits. BMC Musculoskelet Disord.

[59] Gill TJ, Mcculloch PC, Glasson SS, Blanchet T, Morris EA (2005). Chondral defect repair after the microfracture procedure: a nonhuman primate model. Am J Sports Med.

[60] Moseley JB, O’malley K, Petersen NJ, Menke TJ, Brody BA, Kuykendall DH (2002). A controlled trial of arthroscopic surgery for osteoarthritis of the knee. N Engl J Med.

[61] Patel JM, Saleh KS, Burdick JA, Mauck RL (2019). Bioactive factors for cartilage repair and regeneration: improving delivery, retention, and activity. Acta Biomater.

[62] Hunziker E (2002). Articular cartilage repair: basic science and clinical progress. A review of the current status and prospects. Osteoarthr Cartil.

[63] Arslan E, Guler MO, Tekinay AB (2016). Glycosaminoglycan-mimetic signals direct the osteo/chondrogenic differentiation of mesenchymal stem cells in a three-dimensional peptide nanofiber extracellular matrix mimetic environment. Biomacromolecules..

[64] Hutmacher DW. Scaffolds in tissue engineering bone and cartilage. The Biomaterials: Silver Jubilee Compendium: Elsevier; 2000. p. 175–89. 10.1016/s0142-9612(00)00121-6 11071603

[65] Bružauskaitė I, Bironaitė D, Bagdonas E, Bernotienė EJC (2016). Scaffolds and cells for tissue regeneration: different scaffold pore sizes—different cell effects. Cytotechnology..

[66] Beswick AD, Wylde V, Gooberman-Hill R, Blom A, Dieppe P (2012). What proportion of patients report long-term pain after total hip or knee replacement for osteoarthritis? A systematic review of prospective studies in unselected patients. BMJ Open.

[67] Culliford D, Maskell J, Kiran A, Judge A, Javaid M, Cooper C (2012). The lifetime risk of total hip and knee arthroplasty: results from the UK general practice research database. Osteoarthr Cartil.

[68] Caramés B, Taniguchi N, Otsuki S, Blanco FJ, Lotz M (2010). Autophagy is a protective mechanism in normal cartilage, and its aging-related loss is linked with cell death and osteoarthritis. Arthritis Rheum.

[69] Sasaki H, Takayama K, Matsushita T, Ishida K, Kubo S, Matsumoto T (2012). Autophagy modulates osteoarthritis-related gene expression in human chondrocytes. Arthritis Rheum.

[70] Zhang Y, Vasheghani F (2015). Li Y-h, Blati M, Simeone K, Fahmi H, et al. Cartilage-specific deletion of mTOR upregulates autophagy and protects mice from osteoarthritis. Ann Rheum Dis.

[71] Zhen G, Wen C, Jia X, Li Y, Crane JL, Mears SC (2013). Inhibition of TGF-β signaling in mesenchymal stem cells of subchondral bone attenuates osteoarthritis. Nat Med.

[72] Long D, Ulici V, Chubinskaya S, Loeser R (2015). Heparin-binding epidermal growth factor-like growth factor (HB-EGF) is increased in osteoarthritis and regulates chondrocyte catabolic and anabolic activities. Osteoarthr Cartil.

[73] Ornitz DM, Itoh N (2015). The fibroblast growth factor signaling pathway. Wiley Interdiscip Rev Dev Biol.

[74] Bouaziz W, Sigaux J, Modrowski D, Devignes C-S, Funck-Brentano T, Richette P (2016). Interaction of HIF1α and β-catenin inhibits matrix metalloproteinase 13 expression and prevents cartilage damage in mice. Proc Natl Acad Sci.

[75] Henrotin Y, Lambert C, Couchourel D, Ripoll C, Chiotelli E (2011). Nutraceuticals: do they represent a new era in the management of osteoarthritis?–a narrative review from the lessons taken with five products. Osteoarthr Cartil.

[76] Liu X, Eyles J, McLachlan AJ, Mobasheri A (2018). Which supplements can I recommend to my osteoarthritis patients?. Rheumatology.

[77] Goggs R, Vaughan-Thomas A, Clegg PD, Carter SD, Innes JF, Mobasheri A (2005). Nutraceutical therapies for degenerative joint diseases: a critical review. Crit Rev Food Sci Nutr.

[78] Guan VX, Mobasheri A, Probst YC (2019). A systematic review of osteoarthritis prevention and management with dietary phytochemicals from foods. Maturitas.

[79] Henrotin Y, Mobasheri A (2018). Natural products for promoting joint health and managing osteoarthritis. Curr Rheumatol Rep.

[80] Henrotin Y, Mobasheri A, Marty M (2012). Is there any scientific evidence for the use of glucosamine in the management of human osteoarthritis?. Arthritis Res Ther.

[81] Thomas S, Browne H, Mobasheri A, Rayman MP (2018). What is the evidence for a role for diet and nutrition in osteoarthritis?. Rheumatology.

[82] Guermazi A, Roemer FW, Burstein D, Hayashi D (2011). Why radiography should no longer be considered a surrogate outcome measure for longitudinal assessment of cartilage in knee osteoarthritis. Arthritis Res Ther.

[83] Wenham C, Grainger A, Conaghan P (2014). The role of imaging modalities in the diagnosis, differential diagnosis and clinical assessment of peripheral joint osteoarthritis. Osteoarthr Cartil.

[84] Hirvasniemi J, Kulmala K, Lammentausta E, Ojala R, Lehenkari P, Kamel A (2013). In vivo comparison of delayed gadolinium-enhanced MRI of cartilage and delayed quantitative CT arthrography in imaging of articular cartilage. Osteoarthr Cartil.

[85] Weissleder RJS (2006). Molecular imaging in cancer. Science..

[86] Jun YW, Lee JH, Cheon JJACIE (2008). Chemical design of nanoparticle probes for high-performance magnetic resonance imaging. Angew Chem Int Ed.

[87] Waters EA, Wickline SA, Bric J (2008). Contrast agents for MRI. Basic Res Cardiol.

[88] Peng X-H, Qian X, Mao H, Wang AY (2008). Targeted magnetic iron oxide nanoparticles for tumor imaging and therapy. Int J Nanomedicine.

[89] Hashim Z, Green M, Chung PH, Suhling K, Protti A, Phinikaridou A (2014). Gd-containing conjugated polymer nanoparticles: bimodal nanoparticles for fluorescence and MRI imaging. Nanoscale..

[90] Yarmola EG, Kaufman ZA, Arnold DP, Shah Y, Kozissnik B, Garraud A (2014). Probing osteoarthritis biomarkers with magnetic nanoparticles. Biophys J.

[91] Steinert AF, Ghivizzani SC, Rethwilm A, Tuan RS, Evans CH, Nöth UJ (2007). Major biological obstacles for persistent cell-based regeneration of articular cartilage. Arthritis Res Ther.

[92] Koga H, Engebretsen L, Brinchmann JE, Muneta T, Sekiya IJKS (2009). Sports traumatology, arthroscopy. Mesenchymal stem cell-based therapy for cartilage repair: a review. Knee Surg Sports Traumatol Arthrosc.

[93] Wakitani S, Imoto K, Yamamoto T, Saito M, Murata N, Yoneda MJO (2002). Human autologous culture expanded bone marrow mesenchymal cell transplantation for repair of cartilage defects in osteoarthritic knees. Osteoarthr Cartil.

[94] Brittberg M, Lindahl A, Nilsson A, Ohlsson C, Isaksson O, Peterson LJ (1994). Treatment of deep cartilage defects in the knee with autologous chondrocyte transplantation. N Engl J Med.

[95] Niemeyer P, Pestka JM, Kreuz PC, Erggelet C, Schmal H, Suedkamp NP (2008). Characteristic complications after autologous chondrocyte implantation for cartilage defects of the knee joint. Am J Sports Med.

[96] Nedopil A, Klenk C, Kim C, Liu S, Wendland M, Golovko D (2010). MR signal characteristics of viable and apoptotic human mesenchymal stem cells in matrix-associated stem cell implants for treatment of osteoarthritis. Investig Radiol.

[97] Nejadnik H, Ye D, Lenkov OD, Donig JS, Martin JE, Castillo R (2015). Magnetic resonance imaging of stem cell apoptosis in arthritic joints with a caspase activatable contrast agent. ACS Nano.

[98] Saltzherr MS, Selles RW, Bierma-Zeinstra SM, Muradin GS, Coert JH, van Neck JW (2014). Metric properties of advanced imaging methods in osteoarthritis of the hand: a systematic review. Ann Rheum Dis.

[99] Wang LV (2009). Multiscale photoacoustic microscopy and computed tomography. Nat Photonics.

[100] Wang LV, Hu S (2012). Photoacoustic tomography: in vivo imaging from organelles to organs. Science.

[101] Rajian JR, Shao X, Chamberland DL, Wang X (2013). Characterization and treatment monitoring of inflammatory arthritis by photoacoustic imaging: a study on adjuvant-induced arthritis rat model. Biomedical Optics Express.

[102] Xu G, Rajian JR, Girish G, Kaplan MJ, Fowlkes JB, Carson PL (2012). Photoacoustic and ultrasound dual-modality imaging of human peripheral joints. J Biomed Opt.

[103] Weber J, Beard PC, Bohndiek SE (2016). Contrast agents for molecular photoacoustic imaging. Nat Methods.

[104] Kim C, Favazza C, Wang LV (2010). In vivo photoacoustic tomography of chemicals: high-resolution functional and molecular optical imaging at new depths. Chem Rev.

[105] Lin J, Wang M, Hu H, Yang X, Wen B, Wang Z (2016). Multimodal-imaging-guided cancer phototherapy by versatile biomimetic theranostics with UV and γ-irradiation protection. Adv Mater.

[106] Chen L, Ji Y, Hu X, Cui C, Liu H, Tang Y (2018). Cationic poly-L-lysine-encapsulated melanin nanoparticles as efficient photoacoustic agents targeting to glycosaminoglycans for the early diagnosis of articular cartilage degeneration in osteoarthritis. Nanoscale..

[107] Lotz M, Martel-Pelletier J, Christiansen C, Brandi ML, Bruyère O, Chapurlat R (2014). Republished: value of biomarkers in osteoarthritis: current status and perspectives. Postgrad Med J.

[108] Mobasheri A, van Spil WE, Budd E, Uzieliene I, Bernotiene E, Bay-Jensen A-C (2019). Molecular taxonomy of osteoarthritis for patient stratification, disease management and drug development: biochemical markers associated with emerging clinical phenotypes and molecular endotypes. Curr Opin Rheumatol.

[109] Punzi L, Frigato M, Frallonardo P, Ramonda R (2010). Inflammatory osteoarthritis of the hand. Best Pract Res Clin Rheumatol.

[110] Ehrlich GE (2001). Erosive osteoarthritis: presentation, clinical pearls, and therapy. Curr Rheumatol Rep.

[111] Dam EB, Loog M, Christiansen C, Byrjalsen I, Folkesson J, Nielsen M (2009). Identification of progressors in osteoarthritis by combining biochemical and MRI-based markers. Arthritis research & therapy.

[112] Garnero P, Piperno M, Gineyts E, Christgau S, Delmas P, Vignon E (2001). Cross sectional evaluation of biochemical markers of bone, cartilage, and synovial tissue metabolism in patients with knee osteoarthritis: relations with disease activity and joint damage. Ann Rheum Dis.

[113] Van Spil W, Jansen N, Bijlsma J, Reijman M, DeGroot J, Welsing P (2012). Clusters within a wide spectrum of biochemical markers for osteoarthritis: data from CHECK, a large cohort of individuals with very early symptomatic osteoarthritis. Osteoarthr Cartil.

[114] Felson DT, Lawrence RC, Dieppe PA, Hirsch R, Helmick CG, Jordan JM (2000). Osteoarthritis: new insights. Part 1: the disease and its risk factors. Ann Intern Med.

[115] Cowman MK, Lee H-G, Schwertfeger KL, McCarthy JB, Turley EA (2015). The content and size of hyaluronan in biological fluids and tissues. Front Immunol.

[116] Nakamura K, Yokohama S, Yoneda M, Okamoto S, Tamaki Y, Ito T (2004). High, but not low, molecular weight hyaluronan prevents T cell-mediated liver injury by reducing proinflammatory cytokines in mice. J Gastroenterol.

[117] Rayahin JE, Buhrman JS, Zhang Y, Koh TJ, Gemeinhart RA (2015). High and low molecular weight hyaluronic acid differentially influence macrophage activation. ACS Biomater Sci Eng.

[118] Atkinson AJ, Colburn WA, DeGruttola VG, DeMets DL, Downing GJ, Group BDW (2001). Biomarkers and surrogate endpoints: preferred definitions and conceptual framework. Clin Pharmacol Ther.

[119] Li S, Cong W, Hakamivala A, Huang Y, Borrelli J, Tang L (2018). Hyaluronic acid-based optical probe for the diagnosis of human osteoarthritic cartilage. Nanotheranostics..

[120] Rivas F, Zahid OK, Reesink HL, Peal BT, Nixon AJ, DeAngelis PL (2018). Label-free analysis of physiological hyaluronan size distribution with a solid-state nanopore sensor. Nat Commun.

[121] Pratta M, Su J, Leesnitzer M, Struglics A, Larsson S, Lohmander L (2006). Development and characterization of a highly specific and sensitive sandwich ELISA for detection of aggrecanase-generated aggrecan fragments. Osteoarthr Cartil.

[122] Tortorella M, Malfait A-M, Deccico C, Arner E (2001). The role of ADAM-TS4 (aggrecanase-1) and ADAM-TS5 (aggrecanase-2) in a model of cartilage degradation. Osteoarthr Cartil.

[123] Peng S, Zheng Q, Zhang X, Dai L, Zhu J, Pi Y (2013). Detection of ADAMTS-4 activity using a fluorogenic peptide-conjugated Au nanoparticle probe in human knee synovial fluid. ACS Appl Mater Interfaces.

[124] Melchiorri C, Meliconi R, Frizziero L, Silvestri T, Pulsatelli L, Mazzetti I (1998). Enhanced and coordinated in vivo expression of inflammatory cytokines and nitric oxide synthase by chondrocytes from patients with osteoarthritis. Arthritis Rheum.

[125] Amin AR, Di Cesare P, Vyas P, Attur M, Tzeng E, Billiar TR (1995). The expression and regulation of nitric oxide synthase in human osteoarthritis-affected chondrocytes: evidence for up-regulated neuronal nitric oxide synthase. J Exp Med.

[126] Santoro A, Conde J, Scotece M, Abella V, López V, Pino J (2015). Choosing the right chondrocyte cell line: Focus on nitric oxide. J Orthop Res.

[127] Jin P, Wiraja C, Zhao J, Zhang J, Zheng L, Xu CJ (2017). Nitric oxide nanosensors for predicting the development of osteoarthritis in rat model. ACS Appl Mater Interfaces.

[128] Elghanian R, Storhoff JJ, Mucic RC, Letsinger RL, Mirkin CA (1997). Selective colorimetric detection of polynucleotides based on the distance-dependent optical properties of gold nanoparticles. Science..

[129] Wu C-W, Chiang C-Y, Chen C-H, Chiang C-S, Wang C-T, Chau L-K (2016). Self-referencing fiber optic particle plasmon resonance sensing system for real-time biological monitoring. Talanta..

[130] Jeon J, Uthaman S, Lee J, Hwang H, Kim G, Yoo PJ (2018). In-direct localized surface plasmon resonance (LSPR)-based nanosensors for highly sensitive and rapid detection of cortisol. Sensors Actuators B Chem.

[131] Hao D, Hu C, Grant J, Glidle A, Cumming DR (2018). Hybrid localized surface plasmon resonance and quartz crystal microbalance sensor for label free biosensing. Biosens Bioelectron.

[132] Chiang C-Y, Hsieh M-L, Huang K-W, Chau L-K, Chang C-M, Lyu S-R (2010). Fiber-optic particle plasmon resonance sensor for detection of interleukin-1β in synovial fluids. Biosens Bioelectron.

[133] Huang Y-C, Chiang C-Y, Li C-H, Chang T-C, Chiang C-S, Chau L-K (2013). Quantification of tumor necrosis factor-α and matrix metalloproteinases-3 in synovial fluid by a fiber-optic particle plasmon resonance sensor. Analyst..

[134] Jiang T, Kan H-M, Rajpura K, Carbone EJ, Li Y, Lo KW-H (2018). Development of targeted nanoscale drug delivery system for osteoarthritic cartilage tissue. J Nanosci Nanotechnol.

[135] McMasters J, Poh S, Lin JB, Panitch A (2017). Delivery of anti-inflammatory peptides from hollow PEGylated poly (NIPAM) nanoparticles reduces inflammation in an ex vivo osteoarthritis model. J Control Release.

[136] Soliman MS, Abd-Allah FI, Hussain T, Saeed NM, El-Sawy HS (2018). Date seed oil loaded niosomes: development, optimization and anti-inflammatory effect evaluation on rats. Drug Dev Ind Pharm.

[137] Valenti MT, Mottes M, Biotti A, Perduca M, Pisani A, Bovi M (2017). Clodronate as a therapeutic strategy against osteoarthritis. Int J Mol Sci.

[138] Zavgorodnya O, Carmona-Moran CA, Kozlovskaya V, Liu F, Wick TM, Kharlampieva E (2017). Temperature-responsive nanogel multilayers of poly (N-vinylcaprolactam) for topical drug delivery. J Colloid Interface Sci.

[139] Sütő B, Berkó S, Kozma G, Kukovecz Á, Budai-Szűcs M, Erős G (2016). Development of ibuprofen-loaded nanostructured lipid carrier-based gels: characterization and investigation of in vitro and in vivo penetration through the skin. Int J Nanomedicine.

[140] Agarwal R, Volkmer TM, Wang P, Lee LA, Wang Q, García AJ (2016). Synthesis of self-assembled IL-1Ra-presenting nanoparticles for the treatment of osteoarthritis. J Biomed Mater Res A.

[141] Dwivedi P, Nayak V, Kowshik M (2015). Role of gold nanoparticles as drug delivery vehicles for chondroitin sulfate in the treatment of osteoarthritis. Biotechnol Prog.

[142] Jain A, Mishra SK, Vuddanda PR, Singh SK, Singh R, Singh S (2014). Targeting of diacerein loaded lipid nanoparticles to intra-articular cartilage using chondroitin sulfate as homing carrier for treatment of osteoarthritis in rats. Nanomedicine.

[143] Jain A, Singh SK, Singh Y, Singh S (2013). Development of lipid nanoparticles of diacerein, an antiosteoarthritic drug for enhancement in bioavailability and reduction in its side effects. J Biomed Nanotechnol.

[144] Bartlett RL, Sharma S, Panitch A (2013). Cell-penetrating peptides released from thermosensitive nanoparticles suppress pro-inflammatory cytokine response by specifically targeting inflamed cartilage explants. Nanomedicine.

[145] Zhang Z, Leong DJ, Xu L, He Z, Wang A, Navati M (2016). Curcumin slows osteoarthritis progression and relieves osteoarthritis-associated pain symptoms in a post-traumatic osteoarthritis mouse model. Arthritis Res Ther.

[146] Lin JB, Poh S, Panitch A (2016). Controlled release of anti-inflammatory peptides from reducible thermosensitive nanoparticles suppresses cartilage inflammation. Nanomedicine.

[147] Zille H, Paquet J, Henrionnet C, Scala-Bertola J, Leonard M, Six JL (2010). Evaluation of intra-articular delivery of hyaluronic acid functionalized biopolymeric nanoparticles in healthy rat knees. Biomed Mater Eng.

[148] Butoescu N, Seemayer CA, Foti M, Jordan O, Doelker E (2009). Dexamethasone-containing PLGA superparamagnetic microparticles as carriers for the local treatment of arthritis. Biomaterials..

[149] Niazvand F, Khorsandi L, Abbaspour M, Orazizadeh M, Varaa N, Maghzi M, et al., editors. Curcumin-loaded poly lactic-co-glycolic acid nanoparticles effects on mono-iodoacetate-induced osteoarthritis in rats. Veterinary Research Forum; 2017: Faculty of Veterinary Medicine, Urmia University, Urmia. PMC5524554 28785392

[150] Yin L, Agustinus AS, Yuvienco C, Minashima T, Schnabel NL, Kirsch T (2018). Engineered coiled-coil protein for delivery of inverse agonist for osteoarthritis. Biomacromolecules.

[151] Taneepanichskul S, Niempoog S (2015). Improving of knee osteoarthritic symptom by the local application of ginger extract nanoparticles: a preliminary report with short term follow-up. J Med Assoc Thail.

[152] Rehman M, Asadullah Madni AI, Khan WS, Khan MI, Mahmood MA, Ashfaq M (2015). Solid and liquid lipid-based binary solid lipid nanoparticles of diacerein: in vitro evaluation of sustained release, simultaneous loading of gold nanoparticles, and potential thermoresponsive behavior. Int J Nanomedicine.

[153] Geiger BC, Wang S, Padera RF, Grodzinsky AJ, Hammond PT (2018). Cartilage-penetrating nanocarriers improve delivery and efficacy of growth factor treatment of osteoarthritis. Sci Transl Med.

[154] Maudens P, Seemayer CA, Thauvin C, Gabay C, Jordan O, Allémann E (2018). Nanocrystal–polymer particles: extended delivery carriers for osteoarthritis treatment. Small..

[155] Maudens P, Seemayer CA, Pfefferlé F, Jordan O, Allémann E (2018). Nanocrystals of a potent p38 MAPK inhibitor embedded in microparticles: therapeutic effects in inflammatory and mechanistic murine models of osteoarthritis. J Control Release.

[156] Maudens P, Meyer S, Seemayer CA, Jordan O, Allémann E (2018). Self-assembled thermoresponsive nanostructures of hyaluronic acid conjugates for osteoarthritis therapy. Nanoscale..

[157] Villamagna IJ, Gordon TN, Hurtig MB, Beier F, Gillies ER (2019). Poly (ester amide) particles for controlled delivery of celecoxib. J Biomed Mater Res A.

[158] Mota AH, Direito R, Carrasco MP, Rijo P, Ascensão L, Viana AS (2019). Combination of hyaluronic acid and PLGA particles as hybrid systems for viscosupplementation in osteoarthritis. Intern J Pharm.

[159] Bajpayee AG, Quadir MA, Hammond PT, Grodzinsky AJ (2016). Charge based intra-cartilage delivery of single dose dexamethasone using Avidin nano-carriers suppresses cytokine-induced catabolism long term. Osteoarthr Cartil.

[160] Fan W, Li J, Yuan L, Chen J, Wang Z, Wang Y (2018). Intra-articular injection of kartogenin-conjugated polyurethane nanoparticles attenuates the progression of osteoarthritis. Drug delivery.

[161] Feng N, Yang M, Feng X, Wang Y, Chang F, Ding J (2018). Reduction-responsive polypeptide nanogel for intracellular drug delivery in relieving collagen-induced arthritis. ACS Biomater Sci Eng.

[162] Mwangi TK, Bowles RD, Tainter DM, Bell RD, Kaplan DL, Setton LA (2015). Synthesis and characterization of silk fibroin microparticles for intra-articular drug delivery. Int J Pharm.

[163] Arora R, Kuhad A, Kaur I, Chopra K (2015). Curcumin loaded solid lipid nanoparticles ameliorate adjuvant-induced arthritis in rats. Eur J Pain.

[164] Wang J, Wang X, Cao Y, Huang T, Song D, Tao H (2018). Therapeutic potential of hyaluronic acid/chitosan nanoparticles for the delivery of curcuminoid in knee osteoarthritis and an in vitro evaluation in chondrocytes. Int J Mol Med.

[165] Zhao L, Huang J, Fan Y, Li J, You T, He S (2019). Exploration of CRISPR/Cas9-based gene editing as therapy for osteoarthritis. Ann Rheum Diseases.

[166] Gonzalez-Fernandez T, Kelly DJ, O’Brien FJJAT (2018). Controlled non-viral gene delivery in cartilage and bone repair: current strategies and future directions. Adv Ther.

[167] Chin JS, Chooi WH, Wang H, Ong W, Leong KW, Chew SY (2019). Scaffold-mediated non-viral delivery platform for CRISPR/Cas9-based genome editing. Acta Biomater.

[168] Gonzalez-Fernandez T, Rathan S, Hobbs C, Pitacco P, Freeman F, Cunniffe G (2019). Pore-forming bioinks to enable Spatio-temporally defined gene delivery in bioprinted tissues. J Control Release.

[169] Duan J, Dong J, Zhang T, Su Z, Ding J, Zhang Y (2014). Polyethyleneimine-functionalized iron oxide nanoparticles for systemic siRNA delivery in experimental arthritis. Nanomedicine..

[170] Lu H, Dai Y, Lv L, Zhao H (2014). Chitosan-graft-polyethylenimine/DNA nanoparticles as novel non-viral gene delivery vectors targeting osteoarthritis. PLoS One.

[171] Duan W, Li H (2018). Combination of NF-kB targeted siRNA and methotrexate in a hybrid nanocarrier towards the effective treatment in rheumatoid arthritis. J Nanobiotechnol.

[172] Lu H-D, Zhao H-Q, Wang K, Lv L-L (2011). Novel hyaluronic acid–chitosan nanoparticles as non-viral gene delivery vectors targeting osteoarthritis. Int J Pharm.

[173] Zhang X, Yu C, Zhang C, Tang T, Dai K (2006). Direct chitosan-mediated gene delivery to the rabbit knee joints in vitro and in vivo. Biochem Biophys Res Commun.

[174] Deng R-H, Qiu B, Zhou P-H (2018). Chitosan/hyaluronic acid/plasmid-DNA nanoparticles encoding interleukin-1 receptor antagonist attenuate inflammation in synoviocytes induced by interleukin-1 beta. J Mater Sci Mater Med.

[175] Gonzalez-Fernandez T, Tierney EG, Cunniffe GM, O’Brien FJ, Kelly DJ (2016). Gene delivery of TGF-β3 and BMP2 in an MSC-laden alginate hydrogel for articular cartilage and endochondral bone tissue engineering. Tissue Eng A.

[176] Pi Y, Zhang X, Shao Z, Zhao F, Hu X, Ao Y (2015). Intra-articular delivery of anti-Hif-2α siRNA by chondrocyte-homing nanoparticles to prevent cartilage degeneration in arthritic mice. Gene Ther.

[177] Zhou P-H, Qiu B, Deng R-H, Li H-J, Xu X-F, Shang X-F (2018). Chondroprotective effects of hyaluronic acid-chitosan nanoparticles containing plasmid DNA encoding cytokine response modifier A in a rat knee osteoarthritis model. Cell Physiol Biochem.

[178] Liu J, Jiang T, Li C, Wu Y, He M, Zhao J (2019). Bioconjugated carbon dots for delivery of siTNFα to enhance chondrogenesis of mesenchymal stem cells by suppression of inflammation. Stem Cells Transl Med.

[179] Chen X, Liu Y, Wen Y, Yu Q, Liu J, Zhao Y (2019). A photothermal-triggered nitric oxide nanogenerator combined with siRNA for precise therapy of osteoarthritis by suppressing macrophage inflammation. Nanoscale.

[180] Sacchetti C, Liu-Bryan R, Magrini A, Rosato N, Bottini N, Bottini M (2014). Polyethylene-glycol-modified single-walled carbon nanotubes for intra-articular delivery to chondrocytes. ACS Nano.

[181] Simitzi C, Ranella A (2017). Stratakis EJAb. Controlling the morphology and outgrowth of nerve and neuroglial cells: The effect of surface topography. Acta Biomater.

[182] Wu Y-N, Law JBK, He AY, Low HY, Hui JH, Lim CT (2014). Substrate topography determines the fate of chondrogenesis from human mesenchymal stem cells resulting in specific cartilage phenotype formation. Nanomedicine.

[183] Baker BM, Mauck RL (2007). The effect of nanofiber alignment on the maturation of engineered meniscus constructs. Biomaterials..

[184] Mahboudi H, Kazemi B, Soleimani M, Hanaee-Ahvaz H, Ghanbarian H, Bandehpour M (2018). Enhanced chondrogenesis of human bone marrow mesenchymal stem cell (BMSC) on nanofiber-based polyethersulfone (PES) scaffold. Gene..

[185] Alves da Silva M, Martins A, Costa-Pinto A, Costa P, Faria S, Gomes M (2010). Cartilage tissue engineering using electrospun PCL nanofiber meshes and MSCs. Biomacromolecules..

[186] Sonomoto K, Yamaoka K, Kaneko H, Yamagata K, Sakata K, Zhang X (2016). Spontaneous differentiation of human mesenchymal stem cells on poly-lactic-co-glycolic acid nano-fiber scaffold. PLoS One.

[187] Wise JK, Yarin AL, Megaridis CM, Cho M (2008). Chondrogenic differentiation of human mesenchymal stem cells on oriented nanofibrous scaffolds: engineering the superficial zone of articular cartilage. Tissue Eng A.

[188] Xin X, Hussain M, Mao JJJB (2007). Continuing differentiation of human mesenchymal stem cells and induced chondrogenic and osteogenic lineages in electrospun PLGA nanofiber scaffold. Biomaterials..

[189] Wright L, McKeon-Fischer K, Cui Z, Nair L, Freeman J (2014). PDLA/PLLA and PDLA/PCL nanofibers with a chitosan-based hydrogel in composite scaffolds for tissue engineered cartilage. J Tissue Eng Regen Med.

[190] Abedi G, Sotoudeh A, Soleymani M, Shafiee A, Mortazavi P, Aflatoonian MR (2011). A collagen–poly (vinyl alcohol) nanofiber scaffold for cartilage repair. J Biomater Sci Polym Ed.

[191] Arslan E, Ekiz MS, Cimenci CE, Can N, Gemci MH, Ozkan H (2018). Protective therapeutic effects of peptide nanofiber and hyaluronic acid hybrid membrane in in vivo osteoarthritis model. Acta Biomater.

[192] Kim SJ, Kim JE, Kim SH, Kim SJ, Jeon SJ, Kim SH (2016). Therapeutic effects of neuropeptide substance P coupled with self-assembled peptide nanofibers on the progression of osteoarthritis in a rat model. Biomaterials..

[193] Radhakrishnan J, Manigandan A, Chinnaswamy P, Subramanian A, Sethuraman S (2018). Gradient nano-engineered in situ forming composite hydrogel for osteochondral regeneration. Biomaterials..

[194] Hopley EL, Salmasi S, Kalaskar DM, Seifalian AM (2014). Carbon nanotubes leading the way forward in new generation 3D tissue engineering. Biotechnol Adv.

[195] Alice A, Hugh J (2017). Ragione RJJoMCB. Pristine carbon nanotube scaffolds for the growth of chondrocytes. J Mater Chem B.

[196] Chahine NO, Collette NM, Thomas CB, Genetos DC, Loots GG (2014). Nanocomposite scaffold for chondrocyte growth and cartilage tissue engineering: effects of carbon nanotube surface functionalization. Tissue Eng A.

[197] Khang D, Park GE, Webster TJ (2008). Enhanced chondrocyte densities on carbon nanotube composites: the combined role of nanosurface roughness and electrical stimulation. J Biomed Mater Res A.

